# Objective Linguistic Markers Associated with Callous-Unemotional Traits in Early Childhood

**DOI:** 10.1007/s10802-024-01219-4

**Published:** 2024-06-14

**Authors:** R. Waller, M. Flum, Y. Paz, E. R. Perkins, Y. Rodriguez, A. Knox, M.R. Pelella, C. Jones, S. Sun, S.A. Denham, J. Herrington, J. Parish-Morris

**Affiliations:** 1 Department of Psychology, University of Pennsylvania, Philadelphia, PA USA; 2 Center for Autism Research, Children’s Hospital of Philadelphia, Philadelphia, PA USA; 3 George Mason University, Fairfax, VA USA; 4 Department of Child and Adolescent Psychiatry and Behavioral Sciences, Childrens Hospital of Philadelphia, Philadelphia, PA USA; 5 Department of Psychiatry, Perelman School of Medicine, University of Pennsylva, Philadelphia, PA USA

**Keywords:** Callous-unemotional traits, Conduct problems, Computational linguistics, Lymphocyte subsets, Language, Parenting

## Abstract

**Background.** Callous-unemotional (CU) traits are associated with interpersonal difficulties and risk for severe conduct problems (CP). The ability to communicate thoughts and feelings is critical to social success, with language a promising treatment target. However, no prior studies have examined objective linguistic correlates of childhood CU traits in early childhood, which could give insight into underlying risk mechanisms and novel target treatments. **Methods.** We computed lexical (positive emotion, sad, and anger words) and conversational (interruptions and speech rate) markers produced by 131 children aged 5–6 years (*M* = 5.98; *SD* = 0.54, 58.8% female) and their parents while narrating wordless storybooks during two online visits separated by 6–8 weeks (*M* = 6.56, *SD* = 1.11; two books, order counterbalanced). Audio recordings were diarized, time-aligned, and orthographically transcribed using WebTrans. Conversational markers were calculated using R and word frequencies were calculated using Linguistic Inquiry and Word Count (LIWC) software. We examined links between child CU traits and linguistic markers, and explored whether relationships were moderated by child sex. **Results.** Higher CU traits were associated with fewer positive emotion words produced by parents and children. Higher CU traits were also associated with greater concordance in the degree of interruptions and expression of anger emotion words by parents and children. **Conclusions.** Results suggest that objective linguistic correlates of CU traits are detectable during early childhood, which could inform adjunctive treatment modules that improve outcomes by precisely tracking and targeting subtle communication patterns.

**Supplementary Information:**

The online version contains supplementary material available at 10.1007/s10802-024-01219-4.

Conduct problems (CP) are a leading cause of referral to mental health services, resulting in vast public health and economic costs (Goulter et al., 2023). Callous-unemotional (CU) traits (e.g., low empathy and guilt) predict risk for severe CP across development (Frick et al., 2014). Children with CU traits show reduced emotional responsiveness to distress or threat cues (Blair et al., 2014; Northam et al., 2023) and low social affiliation (Viding & McCrory, 2019; Waller & Wagner, 2019). Standard treatments, particularly those targeting parenting, are effective in reducing CP. However, children with CU traits end treatment with greater symptom severity (Perlstein et al., 2023). To improve outcomes, we need novel treatments grounded in a deeper understanding of the socioaffiliative difficulties of children with CU traits.

## Language and CU traits

One way to characterize socioaffiliative difficulties is through language, which is fundamental to human perception, action, and experience (Lindquist et al., 2015), as well as social relationships (Brown et al., 1996; Carpendale & Lewis, 2004). From age 2, children improve dramatically in language comprehension and expression (Grosse et al., 2021; Nook et al., 2017), which contributes to growing social competence (Chow et al., 2021; McCabe & Meller, 2004; Widen, 2013). Notably, children with CU traits have difficulty forming and maintaining social relationships (Chow et al., 2021; Hwang et al., 2022), which may correlate with linguistic challenges. Likewise, children with clinically-significant CP have communicative difficulties that are often overlooked (Dall et al., 2022; Gilmour et al., 2004). The study of language and CU traits is also warranted in the context of research examining language and adult psychopathy. Classic depictions of psychopathy highlight an apparent paradox between a cold and remorseless interpersonal core, alongside a glib, manipulative, or even charming exterior (Cleckley, 1941). Adults with psychopathy are thought to have a fundamental divergence between expression and feeling (Cleckley, 1941; Hare, 1993), seeming to *“know the words but not the music”* (Johns & Quay, 1962). Prior studies show that adults with psychopathy fail to differentiate their emphasis of neutral versus emotional words (Louth et al., 1998) and produce less emotionally intense language (Gullhaugen & Sakshaug, 2019).

However, we lack knowledge about how these linguistic features manifest earlier in development, with only a handful of studies conducted in youth samples. In male offenders aged 13–18 years old, greater verbal skill was linked to more violent offenses for those with high CU traits, but fewer violent offenses for those with low CU traits (Muñoz et al., 2008). Likewise, among adolescent male offenders, better overall pragmatic language based on responses to vignettes on a standardized test was linked to higher CU traits, but only for those without co-occurring anxiety (Anderson et al., 2023). Finally, based on language from autobiographical narratives, adolescent offenders with CU traits expressed more physiological need language (e.g., food, money), but used the second person pronoun, “you”, less than adolescents with low CU traits (Bowman et al., 2023). The takeaway from these studies, mirroring those of adult psychopathy, is that CU traits manifest through restricted expression of interpersonally-relevant language, which is not explained by a general expressive difficulty. This predominance of basic needs or egocentric language may index a tendency to respond to social situations instrumentally or selfishly (Hancock et al., 2013). To improve our understanding of the developmental trajectory of the affective difficulties at the core of psychopathy, studies of language are needed in early childhood, when intervention modules targeting linguistic features could be deployed. In addition, studies need to harness objective approaches to quantify *lexical* and *conversational* features associated with CU traits, including frequency analyses (e.g., counts of specific words) and the dynamics of social interactions (i.e., “back-and-forth” of conversation).

## Lexical Markers

To characterize the lexical features associated with CU traits, a focus on *emotional language* is warranted. Children high on CU traits show limited empathy and prosociality and have difficulties identifying, understanding, and resonating with positive and negative emotional expressions of others (Waller & Wagner, 2019; Waller et al., 2020). In particular, children high on CU traits are worse at recognizing sadness (Hartmann & Schwenck, 2020; Woodworth & Waschbusch, 2008), fail to respond empathically or congruently to sadness (Kimonis et al., 2023; Northam et al., 2023; Paz et al., 2024), and show restricted responses to positive emotion or social bonding cues (O’Nions et al., 2017; Perlstein et al., 2022; Wagner, Waller et al., 2020). At the same time, children high on CU traits show excessive anger towards others (Ciucci et al., 2015; Urben et al., 2017), express anger at inappropriate times (Kimonis et al., 2023), and are often disliked or rejected by peers and teachers (Hwang et al., 2022; Matlasz et al., 2022; Wagner, Bowker et al., 2020). Since language is often central to interactions with others that involve empathy (i.e., sad words), social bonding (i.e., happy words), and conflict (i.e., anger words), an examination of the expression of emotional language may generate insights into the phenomenology and development of CU traits.

## Conversational Markers

In addition to the types of words expressed, interactions are defined by conversational features. Social bonding is strengthened in the context of more turn-taking (Levinson, 2016) and speech alignment (Branigan et al., 2000; Doyle & Frank, 2016; Garrod & Pickering, 2009) between conversational partners. In contrast to the typical turn-taking or speech alignment that define successful social interactions, adults high on psychopathic traits were rated as being more dominant (Kosson et al., 1997) and interrupted more frequently (Manson et al., 2014). Similarly, children with CP and adolescent offenders with psychopathic traits display less hesitation (taken as an index of low reflection and cognitive planning) before responding verbally (Kotsopoulos & Mellor, 1986; Pitchford & Arnell, 2019) and showed greater conversational intrusiveness (e.g., leaning forward and speaking more) (Rime et al., 1978). To date, however, no studies of early childhood have examined conversational features associated with CU traits. Interruptions represent a plausible target of study in light of prior literature focused on psychopathic traits, as well as evidence for social rejection among children with CU traits (i.e., intrusive interruptions perceived as indexing interpersonal dominance; Burgoon et al., 1998; Youngquist, 2009). At the same time, like adults with psychopathy (Cleckley, 1941; Hare, 1993), overall expressivity (i.e., rate of speech) may not be compromised among children with CU or psychopathic traits (Anderson et al., 2022; Muñoz et al., 2008). Thus, alongside emotion words, an examination of interruptions and speech rate, while exploratory, represents a foundational step towards improving our knowledge of why children with CU traits have difficulties forming and maintaining social bonds.

## Parents

Linguistic markers are also relevant for parents, who represent the most important early social influence on young children (Maccoby, 1994). Prior research shows that parenting practices influence the development of CU traits (Waller et al., 2013), including lower parental warmth (i.e., fewer expressions of affection) (Pasalich et al., 2011; Waller et al., 2014) and restricted emotion socialization by parents (i.e., less verbalizing and scaffolding of emotion) (Pasalich et al., 2014) during early and middle childhood. Higher levels of parental harshness (i.e., more expressions of anger or conflict) have also been linked to increases in CU traits across early childhood (Waller et al., 2018). In contrast, greater verbal emotional expression by parents has been linked to higher levels of child empathy, conscientiousness, and prosociality (Brownell et al., 2013; Drummond et al., 2014; Laible, 2004), which converge conceptually with CU traits (Waller et al., 2020). To date, however, no studies have tested whether objectively-quantified linguistic markers produced by parents are related to child CU traits, including by studies that differentiate lexical versus conversational markers. This knowledge could inform the development of novel targets for parent-focused interventions for CP that are tailored to children with CU traits (Fleming et al., 2022; Perlstein et al., 2023).

At the same time, the parent-child relationship is inherently bidirectional. Such reciprocity is established in the study of language and conversational dynamics (Branigan et al., 2000; Doyle & Frank, 2016; Garrod & Pickering, 2009; Group et al., 2009). Successful social bonding and the perception of affiliation during social interactions are contingent on numerous, largely non-conscious, features that signal “togetherness”, including synchrony in eye gaze, facial expressions, and body movements (Prochazkova & Kret, 2017; Van Baaren et al., 2009). The notion of togetherness or bidirectionality is also reflected in studies that have investigated parent-child interactions in relation to child CU traits (Baroncelli & Ciucci, 2020; Hwang et al., 2022; Obando et al., 2023; Waller et al., 2014). However, studies have not investigated whether CU traits are associated with disrupted parent-child reciprocity (i.e., lower concordance) in lexical or conversational features, which represents a significant gap in the literature.

Finally, we know little about sex differences in parent-child communication patterns for children with CU traits. At a population level, it is well established that parents socialize boys and girls differently, including through the language they express (Barnett & Scaramella, 2013; Denham et al., 1994; Leaper et al., 1998), while sex differences in emotional expression emerge in early childhood (Chaplin & Aldao, 2013; Leaper & Smith, 2004). Notably, boys also tend to have higher CU traits (Fontaine et al., 2011) and exhibit greater difficulties recognizing facial expressions of emotion (Winters & Sakai, 2022). Thus, it is plausible that associations between linguistic markers and CU traits differ by sex. However, no studies have investigated sex differences in the associations between CU traits and parent-child language.

In the current study, we adopted a computational linguistics approach to examine how CU traits were related to child and parent *linguistic* (positive, sad, anger emotion words) and *conversational* (e.g., interruptions, speech rate) features. For our first aim, we examined direct associations, hypothesizing that for children and parents, reduced expression of positive and sad emotion words, greater expression of anger words, and more interruptions would be associated with higher child CU traits. We also hypothesized that differences in the expression of emotion words would not be due to overall verbal expressivity (i.e., no association between CU traits and speech rate). For our second aim, we examined the concordance of parent-child language (i.e., correlation between parent and child linguist markers). We hypothesized that positive and sad emotion expression would be less strongly correlated among parent-child dyads for children with high CU traits. In contrast, we hypothesized that interruptions and expression of anger words would be more strongly correlated within parent-child dyads for children with high CU traits, reflective of a more fractious and negative nature of the conversational back-and-forth. For each aim, we examined whether child sex moderated associations between linguistic markers and CU traits or between parent-child linguistic alignment and CU traits, as exploratory hypotheses.

## Methods

### Participants

Participants were 135 children aged 5–6 years (*M* = 5.98; *SD* = 0.54, 58.8% female) and their parents, recruited from a city in the northeastern United States. The majority of parents were biological mothers (96%) who reported being from the following racial groups: White (*n* = 71; 53.0%), Black (*n* = 42; 31.3%, *n* = 1 biracial), Asian (*n* = 18; 13.3%, *n* = 1 biracial), and other/declined to report (*n* = 3; 2.2%). Children were from the following racial groups: White (*n* = 62; 45.9%), Black (*n* = 47; 34.8%, *n* = 4 biracial), Asian (*n* = 17; 12.6%, *n* = 9 biracial), and other (*n* = 9; 6.7%); 6 parents (4.5%) and 9 children (6.5%) also reported being Latino/a/e/x. More than half of parents (52.2%) had a graduate-level degree, 34.5% had a Bachelor level degree, 2.7% had an Associates degree, and 10.7% had a high school degree or less. Average monthly household income was $10,253 (*SD*=$8,742), with 20.2% of the sample reporting an annual income below the area household median based on the 2019 census (U.S. Census Bureau, 2019).

### Procedure

Parents and children took part in two 45-minute Zoom visits separated by 6–8 weeks (*M* = 6.56, *SD* = 1.11), which were recorded. We recruited families through Facebook, flyers posted in community locations, and targeted recruitment through an institutionally-maintained database. Interested families were directed to an online survey asking for basic demographic and contact information followed by a phone screen. Eligible children were 5–6 years old with no learning or developmental disorder diagnosis nor receiving treatment for a psychiatric condition. 395 families expressed interest in participating, 191 completed a phone screen, and 163 were eligible to participate. Of the 163 eligible families, 135 were successfully recruited to the study, which included a baseline assessment (time 1) and follow-up assessment (time 2, *n =* 121; 89.6% retention). Families lost to follow-up did not differ on any study variables or demographic characteristics. Due to attrition and/or technical issues, four families had no language data available at either timepoint, though analyses included families with available language data from at least one timepoint (i.e., *n =* 131; see **Analytic Strategy**).

At time 1, we obtained informed consent from parents (electronic signature) and verbal assent from children. After each visit, parents completed questionnaires through Qualtrics. Families were compensated with Amazon vouchers (time 1, $35; time 2, $45). Between visits, a third of families (*n* = 44) were mailed a social skills game to play four times, a third of families were mailed a mathematics game to play four times (*n* = 44), and a third of families were sent no game (*n* = 43) (Sun et al., 2024). Game assignment was included as a covariate in analyses. Study procedures were approved by the Institutional Review Board at the University of Pennsylvania.

**Fig. 1:**
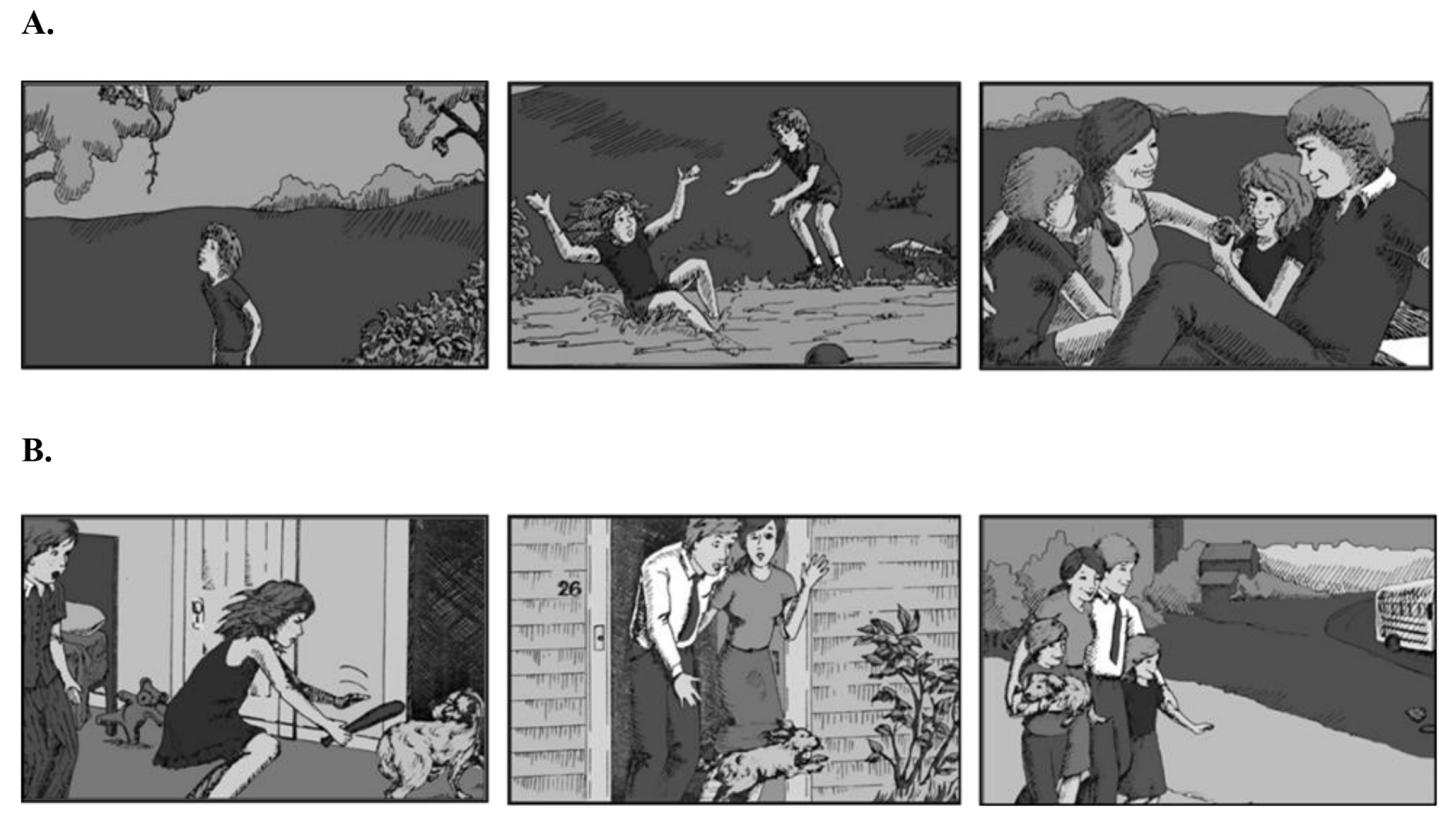
Example images from the two storybook reading tasks, which were completed by families in a counterbalanced order during two online Zoom visits conducted 6–8 weeks apart. **Note. (A)** Three example images from “*The Family Picnic”*: boy loses his kite in a tree, girl slips into the water, family eats lunch together. **(B)** Three example images from *“The Family Pet”*: girl strikes the dog with a baseball bat, the dog runs out of the house, the family goes for a walk together with the dog. Images shown here in greyscale but families saw them in color during the study visit

**Storybook Task.** At time 1 and 2, families read one of two wordless storybooks (Family Picnic and Family Pet; Fig. 1), which were created to elicit emotional language (Garner et al., 2008; Greif et al., 1981), including happiness or excitement (e.g., flying a kite, getting a gift), sadness (e.g., lost ball, hitting a puppy), and anger (e.g., sibling conflict). Storybook order was counterbalanced across time 1 and 2. The research assistant shared their screen and dyads were instructed to “make up the words” to the story with instructions displayed on the screen and read to parents and children before the task began (see Supplemental Materials). Each page was shown for 30 s, after which the next page appeared. Page changes did not occur before 30 s had elapsed, even if participants stopped speaking. We adapted the length of the original stories (Family Pet, 19 pages; Family Picnic, 20 pages) so both took around 10 min to narrate.

### Measures

**Callous-Unemotional Traits.** We measured CU traits using parent report on the Inventory of Callous-Unemotional Traits (ICU; Frick, 2004), a 24-item questionnaire assessing callousness, uncaring, and unemotionality, with items rated on a 4-point scale. Consistent with recommendations (Piacentini et al., 1992) and prior work (Kimonis et al., 2006), we used the higher-rated item from time 1 or 2 (though total ICU scores computed for each time point did not differ when computed separately). While debate remains about the best factor structure for the ICU (Kemp et al., 2022), a common approach is to compute a summed score of 22 or 23 of the 24 items (Ray & Frick, 2020). Similarly, we captured shared variance across items by deriving a latent factor score representing an overarching CU traits construct (α =.84) (Rodriguez et al., 2016). We excluded item 10 (“does not let feelings control him/her’), as higher endorsement was unrelated to other items (i.e., parents interpret as desirable behavior) (Ciucci et al., 2014).

**Covariates.** We controlled for *child sex* (female = 0, male = 1); *child age* in months; *parent education* (less than high school diploma = 1, graduate degree = 6); and *game condition assignment*. We re-ran models for Aim 1 and Aim 2 also controlling for CP to evaluate specificity of findings to CU traits versus general CP severity. We assessed CP using the 5-item CP scale of the parent report Strengths and Difficulties Questionnaire (SDQ; Goodman, 1997) with items rated on a 3-point scale. As before, we used the higher-rated item from time 1 or 2 and derived a latent factor score to capture variance shared by items that represented an overarching CP construct (α = 0.65).

**Data processing**. Audio recordings were orthographically transcribed by annotators who were unaware of study hypotheses. Annotators were undergraduate research assistants trained on a modified Quick Transcription protocol for XTrans software for segmenting and transcription (Cieri et al., 2018; Kimball et al., 2004). Annotators had to exceed 92% word-level reliability criteria before beginning transcription (Parish-Morris et al., 2016). Multiple annotators processed each transcript: the first annotator segmented speech into pause groups (6–8 s long) and labeled each segment as child or parent, with the second and third annotators independently transcribing words. We ran in-house R and Python scripts to identify segments with discrepancies (Cho et al., 2023). Senior annotators (research staff with at least 6 months of XTrans experience) reviewed differences files and adjudicated any discrepancies. After adjudication, files were converted to text format, imported into R, and processed for analysis. Linguistic markers were generated by processing text files using LIWC software (Boyd et al., 2022), which calculates counts for positive emotion, anger, and sad words, analyzed here a percentage of total words spoken. To generate conversational markers, we examined utterances produced by each speaker, until a speaker changed, which were considered one turn. Utterances within turns were defined as speech segments. To assess interruptions, we identified the length of child- or parent-initiated overlapping speech (i.e., negative duration between prior and subsequent speech segments). We included the sum of overlapping speech length in analysis. Overlapping speech was classified as parent- or child-initiated depending on who interrupted the prior speaker, with the sum of interruption duration calculated separately for each. We assessed speech rate (words per minute) by dividing the total number of words by the sum duration of speech segments plus within-turn (i.e., without a speaker change) and between-turn (i.e., with a speaker change) pauses and multiplying this value by 60 (Cho et al., 2023). To generate a robust assessment of lexical and conversational features, we computed mean scores for linguistic markers for the two storybooks.

### Analytic Strategy

Substantive analyses were conducted in Mplus version 7 (Muthén & Muthén, 2017) using full information maximum likelihood estimation with robust standard errors. For our first aim, we examined links between CU traits and either child and parent linguistic markers. Within a single correlated dependent variable model, we simultaneously regressed child positive emotion words, sad words, anger words, interruption, and speech rate onto CU traits, controlling for study condition, child sex, child age, and parent education, while allowing correlation between the linguistic markers. We next ran an identical model but substituted in parent linguistic features. To test for moderation by sex, we ran additional models that included a product term between sex and mean-centered CU traits scores in relation to either the child or parent linguistic markers. For our second aim, we examined the concordance of parent-child linguistic markers, and tested whether concordance varied as a function of CU traits. In separate models, we specified child positive emotion words, sad words, anger words, interruption, and speech rate as the dependent variable. We entered CU traits, the equivalent parent linguistic marker, and the two-way interaction of CU traits and the parent linguistic marker as independent variables, controlling for study condition, child sex, child age, and parent education. Finally, we added two and three-way interaction terms to evaluate whether there were differences in alignment between parent and child linguistic markers as a function of CU traits, sex, or both. Significant interactions were probed using an online tool to generate slopes and regions of significance (www.quantpsy.org).

## Results

Table 1 presents descriptive statistics and Table S1 bivariate correlations between study variables, while Table S2 summarizes child and parent linguistic markers for boys versus girls.

**Table 1:** Descriptive statistics for parent and child linguistic markers and child phenotypic data

Child linguistic markers	M	SD	Min	Max
Total word count	399.06	166.38	15.00	953.00
Speech rate	124.12	17.72	72.66	180.68
Interruption duration	19.76	46.16	0.04	268.32
Positive emotion words	1.16	0.81	0.00	6.67
Sad words	0.65	0.57	0.00	2.92
Anger words	0.80	0.58	0.00	3.05
**Parent linguistic markers**	*M*	*SD*	*Min*	*Max*
Total word count	598.90	264.20	86.00	1227.50
Speech rate	183.72	29.00	108.32	244.37
Interruption duration	42.08	83.24	0.11	484.16
Positive emotion words	1.05	0.49	0.00	3.29
Sad words	0.40	0.34	0.00	2.38
Anger words	0.36	0.38	0.00	2.42
**Child phenotypic data**	*M*	*SD*	*Min*	*Max*
CU traits (latent score)	0.00	0.49	-1.19	1.13
Conduct problems (latent score)	0.03	0.44	-0.46	1.66

Linguistic markers were generated from processing text files in LIWC software (Boyd et al., 2022), which calculates counts for positive emotion, anger, and sad words, presented and analyzed here as a percentage of the total number of words spoken. Conversational markers were derived from utterances produced by each speaker, until a speaker changed, which were considered one turn. Utterances within turns were defined as speech segments. To assess interruptions, we identified overlapping speech (i.e., negative duration between prior and subsequent speech segments). We included the sum of overlapping speech duration (i.e., sum of interruptions) in analysis. We classified overlapping speech as parent or child interruption depending on who was interrupting the previous speaker. We assessed speech rate (number of words per minute) by dividing the total number of words by the sum duration of speech segments plus within-turn (i.e., without a speaker change) and between-turn (i.e., with a speaker change) pause durations and multiplying this value by 60 (Cho et al., 2023). To generate a robust assessment of lexical and conversational features across contexts, we computed mean scores for linguistic markers for children and parents combining across the two storybooks.

### Aim 1: Are CU Traits Directly Related to any Child or Parent Linguistic Markers?

CU traits were associated with lower child expression of positive emotion words (*β=-*0.16, *p =*.04), and marginally related to children interrupting more (*β =* 0.15 *p =*.08). CU traits were not related to children’s expression of sad or anger words, nor their speech rate. Younger children expressed fewer sad words, older children spoke at a faster rate, and girls interrupted more (Table 2). Estimates were similar in magnitude when we included CP as a covariate (*p =*.05; Table S3) and when only child age and sex were included as covariates (*p =*.06; Table S4). There was minimal evidence for moderation by sex (Table S5), with the exception of the association between CU traits and positive emotion word expression (*β=-*0.33, *p =*.01), such that higher CU traits were significantly related to lower positive emotion words expression among girls (B=-0.48, SE = 0.16, *β=-*0.27, *p <*.001) but not boys (B = 0.23, SE = 0.19 *β =.*16, *p =*.22).

**Table 2:** Associations between child CU traits and child and parent linguistic markers

	**Model 1: Child linguistic markers (correlated dependent variables)**																			
	*Interruption*				*Speech Rate*				*Positive emotion words*				*Sad words*				*Anger words*			
	*B*	*SE*	*β*	*p*	*B*	*SE*	*β*	*p*	*B*	*SE*	*β*	*p*	*B*	*SE*	*β*	*p*	*B*	*SE*	*β*	*p*
Study condition	1.29	5.51	0.02	0.81	− 0.23	2.00	− 0.01	0.91	− 0.13	0.07	− 0.13	0.11	− 0.08	0.06	− 0.11	0.18	− 0.05	0.06	− 0.07	0.41
Child sex	**25.20**	**8.26**	**0.27**	**< 0.001**	3.36	3.22	0.09	0.30	0.11	0.12	0.07	0.37	0.05	0.09	0.04	0.58	0.01	0.12	0.01	0.93
Child age	12.19	7.76	0.14	0.08	**7.41**	**3.27**	**0.23**	**0.02**	− 0.26	0.18	− 0.18	0.07	**− 0.23**	**0.09**	**− 0.23**	**0.01**	− 0.09	0.10	− 0.09	0.35
Parent education	4.23	3.60	0.11	0.22	− 0.12	1.71	− 0.01	0.94	− 0.02	0.12	− 0.02	0.90	0.03	0.05	0.06	0.54	0.01	0.05	0.02	0.85
CU traits	13.65	8.21	0.15	0.08	0.11	3.00	0.003	0.97	**− 0.26**	**0.14**	**− 0.16**	**0.04**	− 0.09	0.10	− 0.08	0.37	− 0.09	0.10	− 0.08	0.35
	**Model 2: Parent linguistic markers (correlated dependent variables)**																			
	*Interruption*				*Speech Rate*				*Positive emotion words*				*Sad words*				*Anger words*			
	*B*	*SE*	*β*	*p*	*B*	*SE*	*β*	*p*	*B*	*SE*	*β*	*p*	*B*	*SE*	*β*	*p*	*B*	*SE*	*β*	*p*
Study condition	-1.47	7.57	− 0.01	0.85	0.19	3.11	0.01	0.95	0.01	0.06	0.01	0.90	0.01	0.03	0.01	0.87	− 0.04	0.04	− 0.07	0.39
Child sex	5.82	14.78	0.04	0.70	1.82	5.31	0.03	0.73	0.07	0.10	0.07	0.50	− 0.03	0.05	− 0.05	0.52	0.08	0.06	0.10	0.20
Child age	22.52	14.01	0.15	0.10	2.24	4.76	0.04	0.64	0.02	0.08	0.03	0.76	**− 0.13**	**0.05**	**− 0.21**	**0.02**	− 0.03	0.06	− 0.04	0.66
Parent education	-16.07	8.40	− 0.23	0.05	**5.29**	**2.05**	**0.22**	**0.01**	0.00	0.03	0.01	0.93	− 0.05	0.04	− 0.17	0.18	0.00	0.04	0.01	0.92
CU traits	14.75	16.32	0.09	0.39	0.44	4.73	0.01	0.93	**− 0.21**	**0.09**	**− 0.21**	**0.01**	− 0.10	0.07	− 0.15	0.10	− 0.04	0.07	− 0.05	0.59

Note. For Model 1, all child linguistic markers were included as correlated dependent variables in a single model (see Table S2). For Model 2, all parent linguistic markers were included as correlated dependent variables in a single model (see Table S2).

Higher CU traits in children were also related to lower parent expression of positive emotion words (*β=-*0.21, *p =*.01). CU traits were not related to parent expression of anger and sad words nor their interruptions or speech rate. Parents with higher educational attainment spoke faster and interrupted marginally less, parents of younger children expressed fewer sad words, and parents of girls expressed more anger words (Table 2). Estimates were similar in magnitude including CP as a covariate (*p =*.07; Table S3) and when only child age and sex were covariates (*p =*.01; Table S4). There was minimal evidence for moderation by sex (Table S5), with the exception of the association between CU traits and parent expression of sad words (*β=-*0.36, *p =*.01), such that higher CU traits were significantly related to lower expression of sad words by the parents of girls (*B*=-0.23, *SE* = 0.10, *β=-*0.30, *p <*.01), but not boys (*B* = 0.09, *SE* = 0.09 *β =.*16, *p =*.29).

### Aim 2: Do CU Traits Moderate the Concordance of Parent-Child Linguistic Markers?

**Fig. 2:**
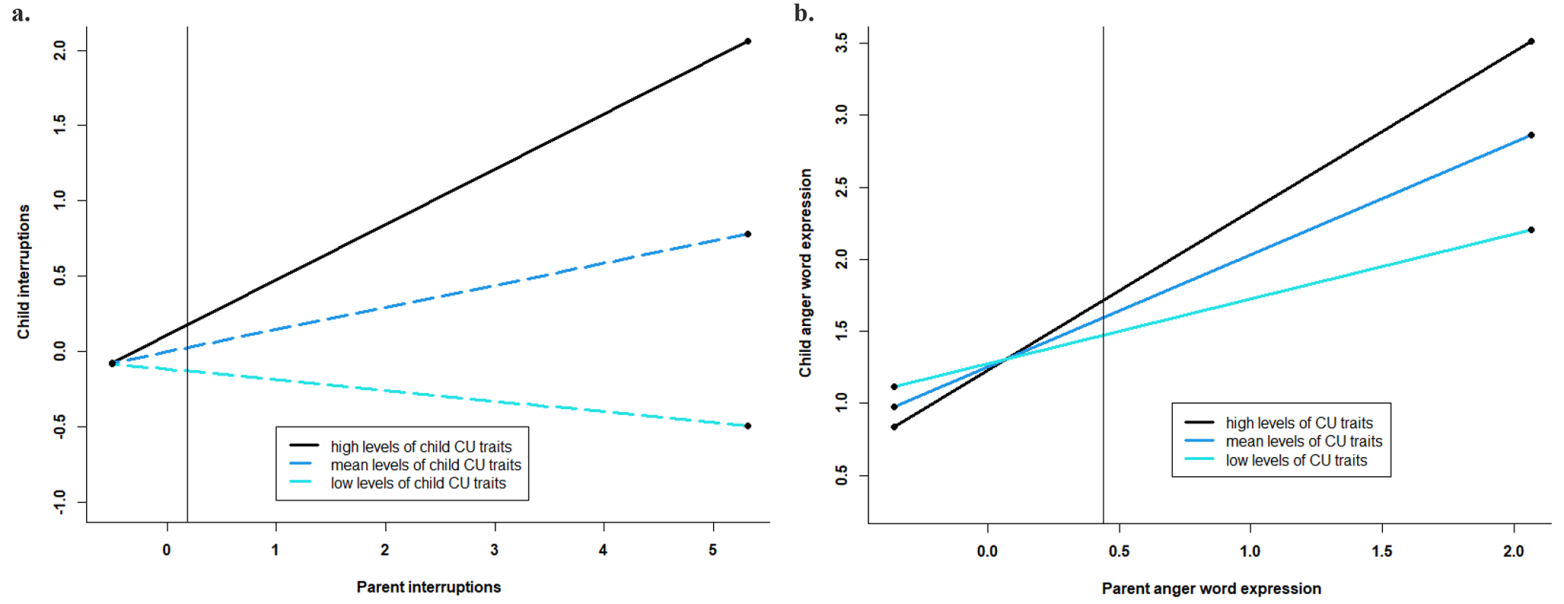
Child CU traits moderate the concordance of interruptions and expression of anger words between parents and children. ***Note.*a.** Child CU traits moderated the degree of parent-child concordance for interruptions, with degree of interruption correlated only among children with high (B = 0.37, SE = 0.10, t = 3.63, *p* <.001), but not mean (B = 0.15, SE = 0.08, t = 1.85, *p* =.07) or low (B=-0.07, SE = 0.11, t=-0.67, *p* =.50). A region of significance analysis indicated that differences between slopes were significant when centered parental interruption values were > 0.19. **b.** Child CU traits moderated the degree of parent-child concordance for anger word expression. Parent-child anger word expression was more strongly correlated among children with high levels of CU traits (B = 1.11, SE = 0.18, t = 6.21, *p* <.001) compared to children with mean (B = 0.78, SE = 0.15, t = 5.30, *p* <.001) or low levels (B = 0.45, SE = 0.18, t = 2.51, *p* =.02) CU traits. A region of significance analysis indicated that the differences between slopes were significant when centered values for parent anger word expression were > 0.44

We found concordance in the speech rate (*β =* 0.23, *p <*.01; Table S6) of parents and children and in their expression of sad (*β =* 0.31, *p <*.001; Table S7) and anger (*β =* 0.44, *p <*.001; Table S8) words, but not interruptions (Table S9) or positive emotion word expression (Table S10). Child CU traits moderated the degree of concordance between parents and children for interruptions (*β =* 0.22, *p =*.01; Table S11) and expression of anger words (*β =* 0.17, *p =.*01; Table S9). Probing these interactions revealed that parent-child interruptions were correlated only when children had high CU traits (*B* = 0.37, *SE* = 0.10, *p* <.001), but not mean (*B* = 0.15, *SE* = 0.08, *p* =.07) or low (*B*=-0.07, *SE* = 0.11, *p* =.50) CU traits (Fig. 2a). Expression of anger words by parents and children was related across the full sample, but the association was stronger for children with high (*B* = 1.11, *SE* = 0.18, *p* <.001) compared to mean (*B* = 0.78, *SE* = 0.15, *p* <.001) or low (*B* = 0.45, *SE* = 0.18, *p* =.02) levels of CU traits. (Fig. 2b). CU traits did not moderate concordance between parents and children in expression of positive emotion or sad words, or speech rate. There was minimal evidence that the degree of concordance between parent-child linguistic markers was moderated by sex, with the exception of anger words (*β=-*0.50, *p =.*04; Table S9). While parent-child anger word expression was related across the sample, the magnitude of the association was larger for boys (*B* = 1.39, *SE* = 0.35, *p <*.001) than girls (*B* = 0.55, *SE* = 0.14, *p <*.001).

## Discussion

We identified a handful of child and parent linguistic markers associated with CU traits in 5- to 6-year-olds, leveraging a naturalistic design where parent-child dyads narrated wordless storybooks. In line with hypotheses, higher CU traits were associated with reduced expression of positive emotion words by parents and children. Findings were similar when controlling for CP, suggestive of a relatively specific association with CU traits, rather than CP severity. A lack of enjoyment and motivation for social closeness with others is theorized as central to the development of CU traits (Viding & McCrory, 2019; Waller & Wagner, 2019). Empirical studies have also linked CU traits to lower social engagement and social imitation in early childhood (Wagner, Waller et al., 2020; Waller et al., 2021). Our results implicate, at least partly, reduced positive emotional expression in this diminished social engagement, which could further reduce the enjoyment experienced by children with CU traits, and their conversational partners, during social interactions (Van Baaren et al., 2009). At the same time, CU traits were unrelated to the expression of other emotion words (i.e., anger, sadness) and speech rate. Here, our results mirror some findings in the adult psychopathy literature suggesting intact verbal expressivity (Gullhaugen & Sakshaug, 2019; Hare, 1993; Louth et al., 1998), even as the emotion behind the words may not be felt to the same degree (Johns & Quay, 1962).

Higher child CU traits also correlated with parents expressing fewer positive emotion words. One explanation for this finding is that parents of children with CU traits share similar affiliative characteristics, consistent with evidence for the heritability of CU traits (Moore et al., 2019). However, parenting practices also impact child CU traits, over and above shared genetic vulnerability (Waller et al., 2018). Thus, our findings may provide an objective, albeit descriptive, linguistic window into the parent-child relationship for children with high CU traits. This interpretation is consistent with evidence that parents of children with CU traits express fewer positive attributions and more negative feelings about their child (Sawrikar et al., 2018) and studies linking lower parental warmth and reciprocity (i.e., fewer verbal expressions of affection or positive emotion) to increases in CU traits over time (Obando et al., 2023; Pasalich et al., 2011; Waller et al., 2014). Importantly, parenting interventions are effective in reducing CU traits, though children with high CU traits still end treatments with greater CP severity than low-CU peers (Perlstein et al., 2023). Our results suggest that increasing positive emotion expression for parents and children could be targeted in adjunctive modules to reduce CU traits (Fleming et al., 2022; Kimonis et al., 2019).

We demonstrated overall concordance between parents and children in their expression of sad words, anger words, and speech rate (though not between positive words or interruptions). These findings are consistent with prior studies showing that social partners tend to align their words and conversational features (Branigan et al., 2000; Doyle & Frank, 2016; Garrod & Pickering, 2009), and that close relationships include social mimicry (Prochazkova & Kret, 2017; Van Baaren et al., 2009). However, parent-child interrupting and the expression of anger words were more strongly correlated when children had high CU traits. These findings could reflect the challenging interpersonal style of high-CU children, objectively documented here through a more fractious or negative conversational back-and-forth (Burgoon et al., 1998; Manson et al., 2014; Youngquist, 2009). Alternatively, more interruption by parents could reflect proactive attempts to scaffold or guide the interaction to redirect negative child behavior. Future studies that use qualitative coding are needed to evaluate the nature of these parent-child effects. Moreover, since we analyzed data at the conversational level, studies are needed to chart dynamics with greater temporal granularity, which can give insight into how CU traits shape the moment-to-moment language expressed during interactions with parents or other social partners.

Finally, consistent with prior research, some child and parent linguistic markers varied as a function of child sex and age (Barnett & Scaramella, 2013; Chaplin & Aldao, 2013; Denham et al., 1994; Foot et al., 1977; Leaper & Smith, 2004). In addition, higher CU traits were related to reduced positive emotion word use by girls and greater sad emotion word use by the parents of girls. In contrast, anger word expression was more strongly aligned between parent-child dyads for boys than girls. Interestingly, studies of language in autism – another condition with pervasive social difficulties – suggest that autism manifests differently in boys and girls, and male-referenced clinical conceptualizations hinder early identification and effective treatment for girls (Boorse et al., 2019; Cho et al., 2023; Parish-Morris et al., 2017). Our results suggest that disruptions in the expression and alignment of linguistic features that contribute to social bonding may relate differently to CU traits as a function of sex. Studies with larger samples are warranted to investigate variations due to sex and age, including in ways that can inform personalized treatment planning.

Our results should be interpreted in light of several limitations. First, we recruited a community sample with low levels of CU traits and CP and high levels of parent education, reducing the generalizability of findings. Second, we aimed to provide a foundational step to inform automated and objective methods to characterize parent-child interactions and generate targets for clinical change for children with CU traits. However, our approach was necessarily exploratory and, with the number of models tested, few findings would survive correction for multiple comparisons. Future research is needed to replicate and extend our findings using larger samples that include families more representative of the general population, and children with clinically-significant CP. Third, we used a storybook task to create an emotional context for parent-child conversations to standardize the elicitation of linguistic markers across families. However, our approach may have reduced ecological validity and the generalizability of findings, including for interpreting the results for interruptions, which could have a different meaning in typical conversational back-and-forth (e.g., “back-channeling”; Gardner, 2001) and in different contexts or cultures ( Group et al., 2009). Moreover, we did not explicitly instruct parents and children to alternate their speech and our operationalization of interruptions is more accurately characterized as a child- or parent-initiated overlapping speech segment. Finally, as data came from Zoom recordings, we could not evaluate other speech variables relevant to psychopathology, including affective prosody and volume alignment (Ding & Zhang, 2023).

We contribute to a growing literature focused on quantifying linguistic features of parents and children. We provide preliminary evidence for links between child CU traits and diminished positive emotion words use by parents and children and negativity in the pattern of parent-child conversational patterns, including more concordant anger and interruption. Our approach highlights the utility of leveraging brief, home-based methods to collect parent-child language samples, thus minimizing demands on families. This study provides a preliminary step towards potential future virtual assessments or adapted interventions for CU traits in early childhood, which could focus on emotion language and other features of parent-child communication.

## Electronic Supplementary Material

Below is the link to the electronic supplementary material.

Supplementary Material 1
