# Supplementary material for: Objective Linguistic Markers Associated with Callous-Unemotional Traits in Early Childhood
Source: Res Child Adolesc Psychopathol. 2024 Jun 14;52(10):1565–76. doi: 10.1007/s10802-024-01219-4 (PMC11461678; doi:10.1007/s10802-024-01219-4)
Supplement: Supplementary file 1 — Supplementary Material 1 [file 10802_2024_1219_MOESM1_ESM.docx]

**Supplemental Materials**

**Objective linguistic markers associated with callous-unemotional traits in early childhood**

**Supplemental Methods**

**Instructions given to parent-child dyads at the start of the storybook reading task.**

**Research Assistant:** *“Time for the last game! You will play this game with your [MOM/DAD]. Are you both ready for the instructions?”*

**Research Assistant ensures that child and parent is ready before moving on.**

**Research Assistant:** *“This is [AMARA]. She really likes to hear stories. For this game, you and your [MOM/DAD] are going to read him a story together. But the problem is that this story doesn’t have any words – only pictures! So for each picture, you and your [MOM/DAD] will have to make up the words.”*

**Research Assistant directing this to the parent:** *“You will have 30 seconds for each page of the book before the next page appears. You should help [CHILD’S NAME] to come up with the story of what’s happening – so you can prompt them and ask questions to help them describe each picture. Do you have any questions?”*

**Research Assistant ensures that child and parent are ready before moving on.**

**First screen is the title of the book.**

**Research Assistant*:****“This is* *the book that you will read to Amara – it’s called,*

***A:*** *“The Family Picnic” by E.B Grief.*

***B:*** *“The Family Pet” but E.B. Grief”*

**Table S1.** Bivariate correlations between continuous study variables

|  | Parent edu | CU traits | CP | Child speech rate | Child inter | Child positive emotion words | Child sad words | Child anger words | Parent speech rate | Parent inter | Parent positive emotion words | Parent sad words |
| --- | --- | --- | --- | --- | --- | --- | --- | --- | --- | --- | --- | --- |
| CU traits | .03 |  |  |  |  |  |  |  |  |  |  |  |
| CP | .004 | .50*** |  |  |  |  |  |  |  |  |  |  |
| Child speech rate | -.08 | -.04 | .07 |  |  |  |  |  |  |  |  |  |
| Child interruption | .04 | .09 | -.02 | .01 |  |  |  |  |  |  |  |  |
| Child positive emotion words | -.03 | -.13 | -.07 | -.33*** | -.05 |  |  |  |  |  |  |  |
| Child sad words | .02 | -.04 | -.16 | -.17 | -.01 | .20* |  |  |  |  |  |  |
| Child anger words | .08 | -.06 | -.13 | -.04 | .11 | .14 | .20* |  |  |  |  |  |
| Parent speech rate | .20* | .004 | -.01 | .21* | .15 | .17 | .17 | .06 |  |  |  |  |
| Parent interruption | -.28** | .06 | .11 | .03 | .13 | .02 | -.15 | -.03 | .001 |  |  |  |
| Parent positive emotion words | -.02 | -.23** | -.14 | .13 | .09 | .05 | -.07 | .05 | .17* | -.10 |  |  |
| Parent sad words | -.12 | -.13 | -.04 | -.08 | .01 | .16 | .32*** | .04 | .05 | -.18* | .05 |  |
| Parent anger words | -.02 | -.05 | -.18* | -.05 | .14 | .003 | -.01 | .50*** | .03 | -.16 | .06 | .25** |
| *Note.* ****p<*.001, ***p<*.01, **p<*.05. Parent edu=parent education. CU traits did not differ significantly between boys (*M*=.08, *SD*=.45) and girls (*M*=-.05, *SD*=.52; *t*=-1.44, *p*=.15). CP were significantly higher in boys (*M*=.11, *SD*=.41) than girls (*M*=-.02, *SD*=.46; *t*=-1.71, *p*=.04). Neither CU traits (*F=*.87, *df=*2, *p=*.42) or CP (*F=*.10, *df=*2, *p=*.90) varied as a function of game condition (i.e., social skills, mathematics, or no game). | | | | | | | | | | | | |

**Table S2.** Paired-sample t-tests comparing parent and child linguistic markers on the basis of sex for combined scores across storybook contexts

|  | **Child linguistic markers** | | | | | **Parent linguistic markers** | | | | |
| --- | --- | --- | --- | --- | --- | --- | --- | --- | --- | --- |
|  | *Boys* | | *Girls* | | *t-test* | *Boys* | | *Girls* | | *t-test* |
|  | *M* | *SD* | *M* | *SD* |  | *M* | *SD* | *M* | *SD* |  |
| Total word count | 384.73 | 163.01 | 409.12 | 169.04 | *t*=.83, *p*=.41 | 621.20 | 252.34 | 583.25 | 272.75 | *t*=-0.81, *p=*.42 |
| Speech rate | 122.74 | 15.55 | 125.09 | 19.13 | *t*=.74, *p*=.46 | 184.44 | 29.29 | 183.22 | 28.97 | *t*=-0.24, *p=*.81 |
| Interruption duration | 8.20 | 23.77 | 27.71 | 55.44 | *t*=.2.41, *p*=.02 | 36.92 | 84.97 | 45.70 | 82.37 | *t*=0.59, *p=*.55 |
| Positive emotion words | 1.06 | 0.62 | 1.24 | 0.92 | *t*=1.22, *p*=.22 | 1.00 | 0.57 | 1.09 | 0.43 | *t*=1.07, *p=*.29 |
| Sad words | 0.60 | 0.49 | 0.68 | 0.62 | *t*=.70, *p*=.49 | 0.39 | 0.27 | 0.41 | 0.38 | *t*=0.37, *p=*.71 |
| Anger words | 0.79 | 0.67 | 0.81 | 0.52 | *t*=.24, *p*=.80 | 0.31 | 0.24 | 0.39 | 0.46 | *t*=1.15, *p=*.25 |

***Note.*** CU traits did not differ significantly between boys (*M*=.08, *SD*=.45) and girls (*M*=-.05, *SD*=.52; *t*=-1.44, *p*=.15). CP were significantly higher in boys (*M*=.11, *SD*=.41) than girls (*M*=-.02, *SD*=.46; *t*=-1.71, *p*=.04).

**Table S3.** Associations between child CU traits and child and parent linguistic markers, controlling for child conduct problems (CP)

|  | **Model 1: Child linguistic markers (correlated dependent variables)** | | | | | | | | | | | | | | | | | | | |
| --- | --- | --- | --- | --- | --- | --- | --- | --- | --- | --- | --- | --- | --- | --- | --- | --- | --- | --- | --- | --- |
|  | *Interruption* | | | | *Speech Rate* | | | | *Positive emotion words* | | | | *Sad words* | | | | *Anger words* | | | |
|  | *B* | *SE* | *β* | *p* | *B* | *SE* | *β* | *p* | *B* | *SE* | *β* | *p* | *B* | *SE* | *β* | *p* | *B* | *SE* | *β* | *p* |
| Study condition | 1.81 | 5.49 | .03 | .74 | -.44 | 2.03 | -.02 | .83 | -.13 | .07 | -.14 | .10 | -.07 | .06 | -.10 | .24 | -.04 | .06 | -.05 | .53 |
| Child sex | 24.59 | 7.83 | .26 | .00 | 3.62 | 3.23 | .10 | .26 | .12 | .12 | .07 | .34 | .04 | .09 | .03 | .67 | .00 | .12 | .00 | 1.00 |
| Child age | 13.05 | 8.40 | .15 | .08 | 7.06 | 3.37 | .22 | .03 | -.27 | .19 | -.18 | .07 | -.22 | .09 | -.21 | .02 | -.08 | .10 | -.08 | .43 |
| Parent education | 4.15 | 3.54 | .11 | .22 | -.09 | 1.71 | -.01 | .96 | -.01 | .12 | -.02 | .90 | .03 | .05 | .06 | .55 | .01 | .05 | .02 | .87 |
| CP | -8.47 | 13.51 | -.08 | .51 | 3.46 | 3.80 | .09 | .36 | .09 | .15 | .05 | .54 | -.16 | .11 | -.13 | .12 | -.14 | .15 | -.11 | .33 |
| CU traits | 17.57 | 12.13 | .19 | .12 | -1.50 | 3.57 | -.04 | .68 | -.30 | .18 | -.18 | .05 | -.02 | .12 | -.02 | .88 | -.03 | .13 | -.02 | .83 |
|  | **Model 2: Parent linguistic markers (correlated dependent variables)** | | | | | | | | | | | | | | | | | | | |
|  | *Interruption* | | | | *Speech Rate* | | | | *Positive emotion words* | | | | *Sad words* | | | | *Anger words* | | | |
|  | *B* | *SE* | *β* | *p* | *B* | *SE* | *β* | *p* | *B* | *SE* | *β* | *p* | *B* | *SE* | *β* | *p* | *B* | *SE* | *β* | *p* |
| Study condition | -2.55 | 7.41 | -.03 | .73 | .27 | 3.09 | .01 | .93 | .01 | .06 | .02 | .86 | .00 | .03 | .01 | .94 | -.03 | .04 | -.05 | .53 |
| Child sex | 7.08 | 14.92 | .04 | .64 | 1.72 | 5.34 | .03 | .75 | .07 | .10 | .07 | .53 | -.03 | .05 | -.05 | .57 | .06 | .06 | .08 | .28 |
| Child age | 2.75 | 14.64 | .14 | .15 | 2.38 | 4.74 | .05 | .62 | .03 | .08 | .03 | .73 | -.14 | .05 | -.22 | .01 | -.01 | .06 | -.01 | .88 |
| Parent education | -15.90 | 8.24 | -.23 | .05 | 5.27 | 2.05 | .22 | .01 | .00 | .04 | .01 | .94 | -.05 | .04 | -.17 | .19 | .00 | .04 | .01 | .95 |
| CP | 17.55 | 19.07 | .09 | .35 | -1.35 | 5.84 | -.02 | .82 | -.05 | .11 | -.04 | .69 | .05 | .09 | .06 | .60 | -.16 | .12 | -.19 | .14 |
| CU traits | 6.62 | 19.18 | .04 | .74 | 1.07 | 5.24 | .02 | .84 | -.19 | .10 | -.19 | .07 | -.13 | .09 | -.18 | .13 | .04 | .09 | .05 | .66 |

**Table S4.** Associations between child CU traits and child and parent linguistic markers, with only child sex and age as covariates

|  | **Model 1: Child linguistic markers (correlated dependent variables)** | | | | | | | | | | | | | | | | | | | |
| --- | --- | --- | --- | --- | --- | --- | --- | --- | --- | --- | --- | --- | --- | --- | --- | --- | --- | --- | --- | --- |
|  | *Interruption* | | | | *Speech Rate* | | | | *Positive emotion words* | | | | *Sad words* | | | | *Anger words* | | | |
|  | *B* | *SE* | *β* | *p* | *B* | *SE* | *β* | *p* | *B* | *SE* | *β* | *p* | *B* | *SE* | *β* | *p* | *B* | *SE* | *β* | *p* |
| Child sex | 22.85 | 7.80 | .24 | .00 | 3.42 | 2.97 | .10 | .25 | .11 | .12 | .07 | .35 | .03 | .09 | .02 | .77 | .00 | .11 | .00 | .99 |
| Child age | 9.85 | 7.23 | .12 | .14 | 7.49 | 2.89 | .23 | .01 | -.25 | .14 | -.17 | .03 | -.25 | .10 | -.24 | .01 | -.10 | .10 | -.09 | .31 |
| CU traits | 13.15 | 8.32 | .14 | .10 | .16 | 2.97 | .00 | .96 | -.23 | .14 | -.14 | .06 | -.08 | .10 | -.07 | .44 | -.09 | .10 | -.07 | .39 |
|  | **Model 2: Parent linguistic markers (correlated dependent variables)** | | | | | | | | | | | | | | | | | | | |
|  | *Interruption* | | | | *Speech Rate* | | | | *Positive emotion words* | | | | *Sad words* | | | | *Anger words* | | | |
|  | *B* | *SE* | *β* | *p* | *B* | *SE* | *β* | *p* | *B* | *SE* | *β* | *p* | *B* | *SE* | *β* | *p* | *B* | *SE* | *β* | *p* |
| Child sex | 15.25 | 15.51 | .09 | .34 | -1.31 | 5.19 | -.02 | .80 | .07 | .09 | .07 | .46 | -.01 | .05 | -.01 | .93 | .07 | .06 | .09 | .21 |
| Child age | 31.31 | 13.70 | .21 | .01 | -.63 | 4.39 | -.01 | .89 | .02 | .08 | .02 | .79 | -.10 | .05 | -.17 | .03 | -.03 | .05 | -.04 | .62 |
| CU traits | 16.26 | 16.36 | .10 | .34 | .00 | 4.91 | .00 | 1.00 | -.21 | .09 | -.21 | .01 | -.10 | .07 | -.15 | .11 | -.03 | .07 | -.04 | .64 |

**Table S5.** Moderation of the associations between child CU traits and child and parent linguistic markers by sex

|  | **Model 1: Child linguistic markers (correlated dependent variables)** | | | | | | | | | | | | | | | | | | | |
| --- | --- | --- | --- | --- | --- | --- | --- | --- | --- | --- | --- | --- | --- | --- | --- | --- | --- | --- | --- | --- |
|  | *Interruption* | | | | *Speech Rate* | | | | *Positive emotion words* | | | | *Sad words* | | | | *Anger words* | | | |
|  | *B* | *SE* | *β* | *p* | *B* | *SE* | *β* | *p* | *B* | *SE* | *β* | *p* | *B* | *SE* | *β* | *p* | *B* | *SE* | *β* | *p* |
| Study condition | 1.37 | 5.52 | .02 | .80 | -.24 | 2.00 | -.01 | .91 | -.13 | .07 | -.13 | .10 | -.08 | .06 | -.11 | .18 | -.05 | .06 | -.07 | .41 |
| Child sex | 24.50 | 8.04 | .26 | .00 | 3.30 | 3.19 | .09 | .30 | .15 | .12 | .09 | .23 | .06 | .09 | .05 | .50 | .02 | .11 | .01 | .89 |
| Child age | 11.48 | 7.80 | .14 | .10 | 7.31 | 3.29 | .23 | .03 | -.22 | .18 | -.15 | .14 | -.22 | .10 | -.22 | .02 | -.09 | .10 | -.08 | .38 |
| Parent edu | 4.04 | 3.63 | .11 | .24 | -.11 | 1.70 | -.01 | .95 | .00 | .12 | .00 | .99 | .03 | .05 | .07 | .48 | .01 | .05 | .02 | .81 |
| CU traits | 6.43 | 7.89 | .07 | .40 | -1.13 | 4.02 | -.03 | .78 | .18 | .21 | .11 | .41 | .02 | .15 | .01 | .92 | -.02 | .17 | -.02 | .91 |
| Child sex x CU traits | 1.52 | 12.63 | .09 | .41 | 1.85 | 5.41 | .04 | .73 | **-.65** | **.24** | **-.32** | **.01** | -.16 | .21 | -.11 | .43 | -.11 | .20 | -.08 | .57 |
|  |  | | | | | | | | | | | | | | | | | | | |
|  | **Model 2: Parent linguistic markers (correlated dependent variables)** | | | | | | | | | | | | | | | | | | | |
|  | *Interruption* | | | | *Speech Rate* | | | | *Positive emotion words* | | | | *Sad words* | | | | *Anger words* | | | |
|  | *B* | *SE* | *β* | *p* | *B* | *SE* | *β* | *p* | *B* | *SE* | *β* | *p* | *B* | *SE* | *β* | *p* | *B* | *SE* | *β* | *p* |
| Study condition | -1.34 | 7.71 | -.01 | .86 | .17 | 3.11 | .01 | .96 | .01 | .06 | .01 | .91 | .00 | .03 | .01 | .89 | -.04 | .04 | -.08 | .39 |
| Child sex | 3.59 | 15.42 | .02 | .82 | 1.92 | 5.33 | .03 | .72 | .08 | .11 | .08 | .48 | -.02 | .05 | -.03 | .74 | .08 | .06 | .11 | .16 |
| Child age | 19.57 | 13.97 | .13 | .16 | 2.40 | 4.77 | .05 | .61 | .03 | .07 | .04 | .65 | -.11 | .05 | -.18 | .04 | -.02 | .06 | -.02 | .77 |
| Parent edu | -17.00 | 8.48 | -.25 | .04 | 5.37 | 2.05 | .22 | .01 | .01 | .04 | .02 | .85 | -.04 | .04 | -.15 | .23 | .01 | .04 | .02 | .86 |
| CU traits | -13.97 | 28.87 | -.08 | .61 | 1.69 | 8.03 | .03 | .83 | -.11 | .20 | -.11 | .58 | .10 | .09 | .15 | .28 | .04 | .08 | .05 | .61 |
| Child sex x CU traits | 43.00 | 33.48 | .21 | .16 | -1.89 | 9.90 | -.03 | .85 | -.15 | .23 | -.12 | .52 | -.31 | .13 | -.36 | .01 | -.12 | .12 | -.12 | .30 |

**Table S6.** Concordance between parent-child speech rate and moderation by CU traits and sex

|  | ***Model 1 – main effect*** | | | | ***Model 2 – moderation by CU traits*** | | | | ***Model 3 – moderation by sex*** | | | |
| --- | --- | --- | --- | --- | --- | --- | --- | --- | --- | --- | --- | --- |
|  | *B* | *SE* | *β* | *p* | *B* | *SE* | *β* | *p* | *B* | *SE* | *β* | *p* |
| Group | -.22 | 1.89 | -.01 | .91 | -.24 | 1.89 | -.01 | .90 | -.12 | 1.92 | -.01 | .95 |
| Child sex | 3.05 | 2.92 | .09 | .30 | 3.04 | 3.02 | .09 | .32 | 3.06 | 2.91 | .09 | .30 |
| Child age | **7.04** | **3.21** | **.22** | **.03** | 6.98 | 3.33 | .22 | .04 | 6.99 | 3.20 | .22 | .03 |
| Parent education | -.95 | 1.83 | -.06 | .60 | -.97 | 1.84 | -.07 | .60 | -.91 | 1.83 | -.06 | .62 |
| Parent speech rate | **.14** | **.05** | **.23** | **<.01** | .14 | .05 | .23 | .00 | .17 | .07 | .27 | .01 |
| CU traits |  |  |  |  | .07 | 3.02 | .00 | .98 |  |  |  |  |
| CU traits x parent speech rate |  |  |  |  | .03 | .10 | .02 | .77 |  |  |  |  |
| Sex x parent speech rate |  |  |  |  |  |  |  |  | -.05 | .09 | -.06 | .63 |
| **R^2^** | **.11*** | | | | **.11*** | | | | **.11*** | | | |
| ***Note****.* In a model that included all relevant two-way interactions, the three-way interaction of sex x CU traits x parent speech rate was not significant. **p<*.05 | | | | | | | | | | | | |

**Table S7.** Concordance between parent-child sad word expression and moderation by CU traits and sex

|  | ***Model 1 – main effect*** | | | | ***Model 2 – moderation by CU traits*** | | | | ***Model 3 – moderation by sex*** | | | |
| --- | --- | --- | --- | --- | --- | --- | --- | --- | --- | --- | --- | --- |
|  | *B* | *SE* | *β* | *p* | *B* | *SE* | *β* | *p* | *B* | *SE* | *β* | *p* |
| Group | -.08 | .06 | -.11 | .15 | -.08 | .06 | -.11 | .16 | -.09 | .06 | -.13 | .12 |
| Child sex | .07 | .09 | .06 | .40 | .10 | .10 | .09 | .28 | .08 | .09 | .07 | .39 |
| Child age | -.16 | .09 | -.16 | .08 | -.14 | .09 | -.13 | .13 | -.17 | .10 | -.17 | .07 |
| Parent education | .06 | .04 | .12 | .18 | .05 | .04 | .11 | .22 | .06 | .04 | .12 | .18 |
| Parent sad words | **.52** | **.14** | **.31** | **<.001** | .65 | .21 | .39 | .00 | .27 | .34 | .16 | .44 |
| CU traits |  |  |  |  | -.04 | .10 | -.03 | .71 |  |  |  |  |
| CU traits x parent sad words |  |  |  |  | .42 | .30 | .15 | .16 |  |  |  |  |
| Sex x parent sad words |  |  |  |  |  |  |  |  | .34 | .38 | .17 | .36 |
| **R^2^** | **.16*** | | | | **.18*** | | | | **.27*** | | | |
| ***Note****.* In a model that included all relevant two-way interactions, the three-way interaction of sex x CU traits x parent speech rate was not significant. **p<*.05 | | | | | | | | | | | | |

**Table S8.** Concordance between parent-child anger word expression and moderation by CU traits and sex

|  | ***Model 1 – main effect*** | | | | ***Model 2 – moderation by CU traits*** | | | | ***Model 3 – moderation by sex*** | | | |
| --- | --- | --- | --- | --- | --- | --- | --- | --- | --- | --- | --- | --- |
|  | *B* | *SE* | *β* | *p* | *B* | *SE* | *β* | *p* | *B* | *SE* | *β* | *p* |
| Group | -.08 | .06 | -.11 | .15 | -.03 | .05 | -.04 | .56 | -.01 | .05 | -.02 | .79 |
| Child sex | .07 | .09 | .06 | .40 | -.03 | .10 | -.03 | .75 | -.05 | .11 | -.04 | .65 |
| Child age | -.16 | .09 | -.16 | .08 | -.06 | .08 | -.06 | .44 | -.04 | .08 | -.04 | .60 |
| Parent education | .06 | .04 | .12 | .18 | -.01 | .04 | -.01 | .87 | .02 | .04 | .05 | .52 |
| Parent anger words | **.52** | **.14** | **.31** | **<.001** | .78 | .15 | .51 | <.001 | 1.39 | .35 | .91 | <.001 |
| CU traits |  |  |  |  | -.05 | .08 | -.04 | .54 |  |  |  |  |
| CU traits x parent anger words |  |  |  |  | **.67** | **.21** | **.21** | **<.001** |  |  |  |  |
| Sex x parent anger words |  |  |  |  |  |  |  |  | **-.84** | **.37** | **-.50** | **.04** |
| **R^2^** | **.20***** | | | | **.25***** | | | | **.25***** | | | |
| ***Note****.* In a model that included all relevant two-way interactions, the three-way interaction of sex x CU traits x parent speech rate was not significant. ****p<*.001 | | | | | | | | | | | | |

**Table S9.** Concordance between parent-child interruptions and moderation by CU traits and sex

|  | ***Model 1 – main effect*** | | | | ***Model 2 – moderation by CU traits*** | | | | ***Model 3 – moderation by sex*** | | | |
| --- | --- | --- | --- | --- | --- | --- | --- | --- | --- | --- | --- | --- |
|  | *B* | *SE* | *β* | *p* | *B* | *SE* | *β* | *p* | *B* | *SE* | *β* | *p* |
| Group | .73 | 5.48 | .01 | .89 | 1.85 | 5.35 | .03 | .73 | 23.10 | 7.75 | .01 | .93 |
| Child sex | 22.83 | 7.72 | .24 | <.001 | 21.51 | 8.33 | .23 | <.001 | 9.13 | 6.79 | .25 | <.001 |
| Child age | 8.82 | 6.91 | .10 | .16 | 7.00 | 7.14 | .08 | .29 | 5.87 | 3.47 | .11 | .13 |
| Parent education | 5.52 | 3.60 | .14 | .11 | 6.15 | 3.22 | .16 | .04 | .05 | .07 | .15 | .08 |
| Parent interruption | .08 | .06 | .14 | .19 | .08 | .04 | .15 | .11 | .04 | .10 | .09 | .46 |
| CU traits |  |  |  |  | 1.99 | 7.28 | .12 | .11 |  |  |  |  |
| CU traits x parent interruption |  |  |  |  | **.25** | **.07** | **.22** | **.01** |  |  |  |  |
| Sex x parent interruption |  |  |  |  |  |  |  |  | 23.10 | 7.75 | .06 | .69 |
| **R^2^** | **.08*** | | | | **.14*** | | | | **.08*** | | | |
| ***Note****.* In a model that included all relevant two-way interactions, the three-way interaction of sex x CU traits x parent speech rate was not significant. **p<*.05 | | | | | | | | | | | | |

**Table S10.** Concordance between parent-child positive emotion word expression and moderation by CU traits and sex

|  | ***Model 1 – main effect*** | | | | ***Model 2 – moderation by CU traits*** | | | | ***Model 3 – moderation by sex*** | | | |
| --- | --- | --- | --- | --- | --- | --- | --- | --- | --- | --- | --- | --- |
|  | *B* | *SE* | *β* | *p* | *B* | *SE* | *β* | *p* | *B* | *SE* | *β* | *p* |
| Group | -.11 | .07 | -.12 | .17 | -.13 | .07 | -.13 | .13 | -.12 | .07 | -.12 | .17 |
| Child sex | .14 | .12 | .09 | .25 | .11 | .12 | .07 | .36 | .14 | .12 | .09 | .25 |
| Child age | -.23 | .18 | -.16 | .11 | -.25 | .18 | -.17 | .09 | -.23 | .18 | -.16 | .11 |
| Parent education | -.02 | .12 | -.02 | .90 | -.02 | .12 | -.02 | .90 | -.01 | .12 | -.02 | .93 |
| Parent positive emotion words | .08 | .11 | .05 | .46 | .05 | .13 | .03 | .71 | .20 | .18 | .12 | .25 |
| CU traits |  |  |  |  | -.25 | .15 | -.15 | .06 |  |  |  |  |
| CU traits x parent positive emotion words |  |  |  |  | .12 | .23 | .04 | .60 |  |  |  |  |
| Sex x parent positive emotion words |  |  |  |  |  |  |  |  | -.26 | .24 | -.10 | .28 |
| **R^2^** | **.05** | | | | **.07** | | | | **.06** | | | |
| ***Note****.* In a model that included all relevant two-way interactions, the three-way interaction of sex x CU traits x parent speech rate was not significant. | | | | | | | | | | | | |
